# Endogenous MOV10 inhibits the retrotransposition of endogenous retroelements but not the replication of exogenous retroviruses

**DOI:** 10.1186/1742-4690-9-53

**Published:** 2012-06-22

**Authors:** Shetal Arjan-Odedra, Chad M Swanson, Nathan M Sherer, Steven M Wolinsky, Michael H Malim

**Affiliations:** 1 Department of Infectious Diseases, King's College London School of Medicine, 2nd Floor, Borough Wing, Guy's Hospital, London Bridge, London, SE1 9RT, UK; 2 Division of Infectious Diseases, Northwestern University Feinberg School of Medicine, Chicago, IL, 60611-2826, USA; 3 Present Address: Institute for Molecular Virology and McArdle Laboratory for Cancer Research, University of Wisconsin-Madison, Madison, WI, 53706, USA

**Keywords:** MOV10, Retrovirus, Retrotransposon, APOBEC3

## Abstract

**Background:**

The identification of cellular factors that regulate the replication of exogenous viruses and endogenous mobile elements provides fundamental understanding of host-pathogen relationships. MOV10 is a superfamily 1 putative RNA helicase that controls the replication of several RNA viruses and whose homologs are necessary for the repression of endogenous mobile elements. Here, we employ both ectopic expression and gene knockdown approaches to analyse the role of human MOV10 in the replication of a panel of exogenous retroviruses and endogenous retroelements.

**Results:**

MOV10 overexpression substantially decreased the production of infectious retrovirus particles, as well the propagation of LTR and non-LTR endogenous retroelements. Most significantly, RNAi-mediated silencing of endogenous MOV10 enhanced the replication of both LTR and non-LTR endogenous retroelements, but not the production of infectious retrovirus particles demonstrating that natural levels of MOV10 suppress retrotransposition, but have no impact on infection by exogenous retroviruses. Furthermore, functional studies showed that MOV10 is not necessary for miRNA or siRNA-mediated mRNA silencing.

**Conclusions:**

We have identified novel specificity for human MOV10 in the control of retroelement replication and hypothesise that MOV10 may be a component of a cellular pathway or process that selectively regulates the replication of endogenous retroelements in somatic cells.

## Background

Exogenous retroviruses and endogenous retroelements replicate in the host by reverse transcribing their RNA genomes into DNA copies that are permanently integrated into the host genome, making them some of the most successful parasites studied. Approximately 45% of the human genome is derived from mobile elements, with active long interspersed nucleotide element-1 (LINE-1), Alu and SINE-R/VNTR/Alu (SVA) retrotransposition events contributing to disease-producing insertional mutations in humans [1-4]. Host cells have evolved multiple transcriptional and post-transcriptional control mechanisms to protect themselves and their genomes from the pathogenic and mutagenic effects of such parasites.

Cellular restriction factors form an effective innate defence against a range of exogenous retroviruses and intracellular retroelements. The human APOBEC3 (*apo*lipoprotein B mRNA-*e*diting enzyme *c*atalytic polypeptide 1-like 3) family of cytidine deaminases are potent intrinsic antiviral factors that restrict a broad range of exogenous retroviruses [5-9] as well as the propagation of numerous endogenous retroelements [6-10]. Similarly, TRIM5α [11], tetherin [12] and SAMHD1 [13,14] are restriction factors that can inhibit the replication of exogenous retroviruses at different steps in the retroviral life cycle [15]. Intriguingly, the cytosolic exonuclease TREX1 metabolises reverse-transcribed DNA derived from endogenous retroelements and, presumably, restricts their retrotransposition [16], yet is a co-factor for human immunodeficiency virus type-1 (HIV-1) infection [17] revealing the complexity of host-pathogen interactions.

MOV10 (Moloney leukaemia virus 10) is a superfamily 1 (SF1) putative RNA helicase that acts as a co-factor or inhibitory factor for a number of RNA viruses. MOV10 is required for the replication of human hepatitis delta virus (HDV) [18] but restricts hepatitis C virus (HCV) and vesicular stomatitis virus (VSV) replication [19,20]. The antiviral function of MOV10 is evolutionarily conserved as its ortholog in *Arabidopsis thaliana*, SDE3 (silencing defective protein 3), regulates small-RNA mediated silencing of specific exogenous viruses [21], whereas its ortholog in *Drosophila melanogaster*, Armitage, and its mammalian paralog, MOV10-like-1 (MOV10L1), are necessary for piRNA-mediated repression of endogenous retroelements [22-26]. MOV10 has also been reported to associate with the RNA-induced silencing complex (RISC) that mediates small RNA-mediated RNA silencing [27]. Recently, we and others identified MOV10 as interacting with the antiviral APOBEC3 proteins, APOBEC3G (A3G) and APOBEC3F (A3F), in an RNA-dependent manner [28,29]. Taken together, these observations suggest that human MOV10 may regulate a wide range of RNA viruses and could also control the retrotransposition of endogenous retroelements in mammals.

Supporting the hypothesis that MOV10 is an antiviral factor, several groups have reported that MOV10 overexpression restricts the infectivity of HIV-1 and other retroviruses [30-33], although the proposed mechanisms of action differ. Endogenous MOV10 is packaged into HIV-1 virions produced from infected monocyte-derived macrophages, and recently it was reported that MOV10 packaging requires the nucleocapsid region of Gag [30,32-34]. Crucially, these reports varied substantially in their conclusions regarding the effect of depleting endogenous MOV10 on HIV-1 replication in that they either observed a slight decrease in infectivity [31], a modest increase in infectivity [32], or a small decrease in virus production with no difference in infectivity [30]. These contrasting results have led to confusion over whether MOV10 is a co-factor or an inhibitory factor for HIV-1 replication. Furthermore, the possible role of MOV10 in regulating the replication of endogenous retroelements in mammalian cells awaits examination.

To define MOV10′s capacity to regulate retroelements, we undertook side-by-side comparisons of the effects of MOV10 overexpression and depletion on the replication of a number of exogenous retroviruses and the retrotransposition of endogenous retroelements. Our results indicate that MOV10 overexpression restricts the production of infectious virions for a broad range of exogenous retroviruses and also potently inhibits the mobilisation of endogenous retroelements. Importantly, silencing of endogenous MOV10 has no effect on the replication of exogenous retroviruses though it significantly enhances the transposition of human endogenous retrotransposons and a mouse endogenous retrovirus. Furthermore, we report that MOV10 is not necessary for miRNA or siRNA-mediated RNA silencing in cultured cells.

## Results

### MOV10 overexpression restricts the production of infectious retrovirus particles

**Figure 1:**
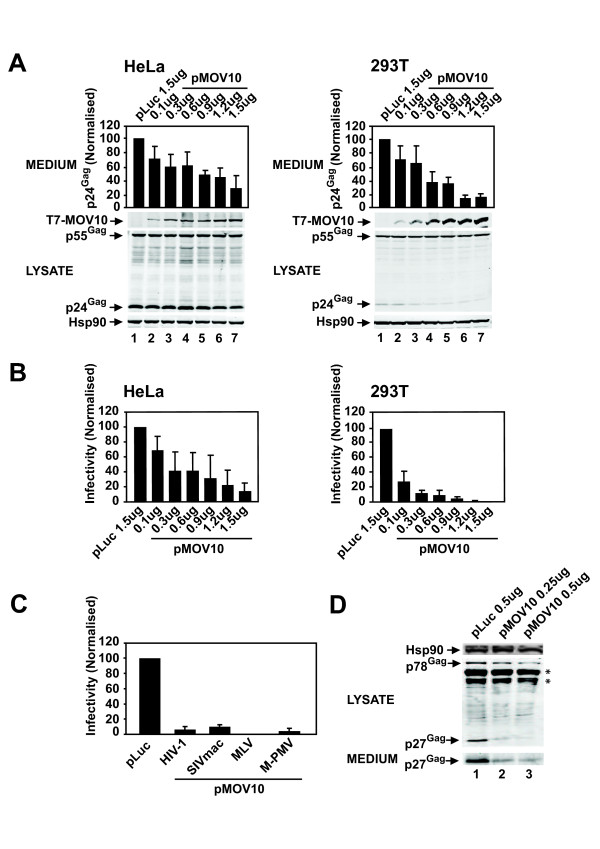
**MOV10 overexpression restricts the production of infectious retrovirus particles for a broad range of exogenous retroviruses. (A)** MOV10 overexpression decreases HIV-1 virus production. HeLa or 293T cells were co- transfected with pHIV-1_NL4-3_ and increasing amounts of pT7-MOV10 as indicated or the pT7-Luc control. Virus concentration in the medium was determined by a p24^Gag^ ELISA. Cell lysates were analysed by quantitative immunoblotting with anti-T7, anti- p24^Gag^ and anti-Hsp90 antibodies. **(B)** Overexpression of MOV10 inhibits the infectivity of HIV-1 virions. The TZM-bl reporter cell line expressing a HIV-1 Tat inducible β-gal reporter gene was infected with equal amounts of virus normalised by the p24^Gag^ concentration from each of the indicated samples. Cells were lysed and β-gal activity was measured to determine virus infectivity. **(C)** MOV10 overexpression inhibits the production of infectious SIVmac, MLV and M-PMV particles. For HIV-1 and SIVmac lentiviral vector production, 293T cells were co-transfected with p8.91, pCSGW and pVSV-G, or pSIV3+, pSIV-RMES4 and pVSV-G, respectively, together with pT7-MOV10 or pT7-Luc. 293T cells were infected with lentiviral particles and infectivity was determined by measuring the percentage of GFP-positive 293T cells by FACS. For MLV and M-PMV virion production, 293T cells were co-transfected with pNCS/FLAG, pMSCV/Tat and pVSV-G, or pMTΔE and pVSV-G, respectively, together with pT7-MOV10 or pT7-Luc. Infectivity was determined using TZM-bl cells. **(D)** Overexpression of MOV10 decreases the production of M-PMV virions. Cell lysates and sucrose cushion purified M-PMV virions were analysed by immunoblotting with anti p27^Gag^ and anti-Hsp90 antibodies (* refers to non-specific bands). For (A), (B) and (C) results are normalised to the pLuc control, which is set at 100%. For (C) a single control bar set at 100% is graphed for simplicity. Values are the mean ± SD of 3 independent experiments**.**

To determine whether the overexpression of MOV10 affects HIV-1 virion production and infectivity, we co-transfected HeLa or 293T cell lines with pHIV-1_NL4-3_[35] and increasing amounts of pMOV10 or a pluciferase (pLuc) control vector (pT7-MOV10 or pT7-Luc). The virion concentration was determined by p24^Gag^ enzyme-linked immunosorbent assay (ELISA). We observed a consistent dose-dependent decrease in the production of virions from HeLa and 293T cells, whereby at the maximum dose of pMOV10 virus production was reduced by ~70% and ~80%, respectively (Figure 1A). We then tested the infectivity of these virions by adding equal amounts of virus normalised by the p24^Gag^ concentration to the TZM-bl reporter cell line. Overexpression of MOV10 decreased the infectivity of HIV-1 virions substantially in a dose-dependent manner, and at the maximum amount of pMOV10 infectivity was reduced by ~80% for HeLa cells and to undetectable levels for 293T cells (Figure 1B). Cell lysates were analysed by immunoblotting to determine whether MOV10 overexpression affected Gag expression or processing. We quantified all the Gag bands to measure total cellular Gag levels and also determined the percentage of Gag processing (total processed Gag bands divided by total Gag bands). Total cellular Gag levels decreased by ~40% and ~50% in HeLa and 293T cells, respectively, at the maximum pMOV10 amount when compared with the pLuc control (Figure 1A, compare lanes 1 and 7). Furthermore, Gag processing was slightly reduced by ~10% and ~40% in HeLa and 293T cells, respectively (Figure 1A, compare lanes 1 and 7). Therefore, the overexpression of MOV10 decreased the production and infectivity of HIV-1 virions in a dose-dependent manner, and also caused a modest decrease in Gag expression and processing.

We then determined whether MOV10 overexpression also restricts the infectivity of a selection of divergent retroviruses including rhesus macaque-derived simian immunodeficiency virus (SIVmac, a lentivirus), murine leukaemia virus (MLV, a gammaretrovirus) and Mason-Pfizer monkey virus (M-PMV, a betaretrovirus). We produced SIVmac vectors by transfecting 293T cells with an SIVmac Gag-Pol packaging plasmid (pSIV3-RMES4) [36], a GFP-expressing SIVmac vector (pSIV-RMES4) [36] and pVSV-G [37]. As a control, we also tested analogous VSV-G pseudotyped HIV-1 vectors, produced using the HIV-1 Gag-Pol plasmid p8.91 [38], the HIV-1 GFP-expressing vector pCSGW [39] and pVSV-G. Plasmids for lentiviral vector production were co-transfected with either pMOV10 or the pLuc control. The effect on the production of infectious particles was determined by challenging 293T cells and measuring the percentage of GFP-positive cells. Similar to the wild-type HIV-1 experiments (Figure 1B), overexpression of MOV10 reduced the production of infectious HIV-1 and SIVmac particles by over 80% relative to the pLuc control (Figure 1C).

To test MLV infectivity, we co-transfected 293T cells with a full-length MLV proviral plasmid (pNCS/FLAG) [40] together with a surrogate MLV genome expressing HIV-1 Tat (pMSCV/Tat) [41] and pVSV-G. To analyse M-PMV infectivity, 293T cells were co-transfected with a M-PMV proviral plasmid in which *env* is replaced with HIV-1 *tat* (pMTΔE) [42] together with pVSV-G. Plasmids for the production of both MLV and M-PMV virions were co-transfected together with either pMOV10 or the pLuc control. The effect on the production of infectious MLV and M-PMV virions was determined by infecting the TZM-bl reporter cells. Overexpression of MOV10 decreased the production of MLV and M-PMV infectious virions by over 80% with respect to the pLuc control (Figure 1C). We also analysed sucrose cushion purified M-PMV virions and cell lysates by immunoblotting to determine the effect of MOV10 overexpression on virus production. Similar to HIV-1 (Figure 1A), we observed a decrease in M-PMV precursor p78^Gag^ and processed p27^Gag^ levels in the cell lysate as well as a decrease in virion production with increasing concentrations of pMOV10 relative to the pLuc control (Figure 1D, compare lane 1 with lanes 2 and 3). These results show that MOV10 overexpression can restrict the production and infectivity of retroviruses from multiple genera.

### Overexpression of MOV10 inhibits the retrotransposition of LTR and non-LTR endogenous retroelements

Similar to exogenous retroviruses, endogenous retroelements replicate via an RNA intermediate that is reverse transcribed and integrated into the host genome. Considering the association of MOV10 homologs with the suppression of endogenous mobile elements [22-24], we next assessed whether overexpression of MOV10 inhibits the retrotransposition of some representative endogenous retroelements. We tested the non-LTR autonomous human LINE-1 and its dependent non-autonomous short interspersed nucleotide element (SINE) Alu retrotransposons, both of which reverse transcribe by target-site primed reverse transcription (TPRT) in the nucleus [1]. We also included the mouse intracisternal A-type particle (IAP), which is related to the betaretrovirus family of exogenous retroviruses, though it has a strictly intracellular life cycle [43].

**Figure 2:**
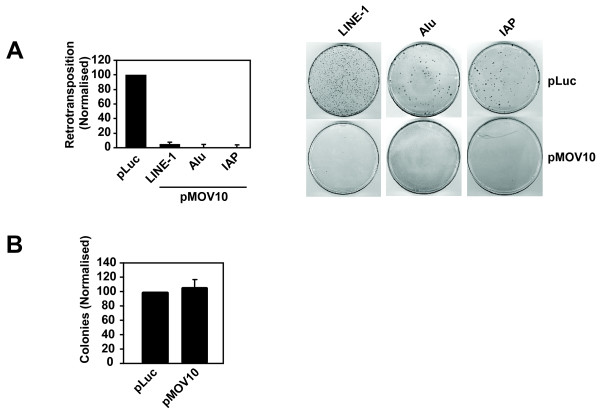
**Overexpression of MOV10 suppresses the retrotransposition of both LTR and non-LTR endogenous retroelements. (A)** MOV10 overexpression restricts the replication of LINE-1, Alu and IAP. HeLa cells were co-transfected with pLINE-1 (pJM101/L1.3), pAlu (pAlu-neo^Tet^ plus pCEP-ORF2), or pIAP (pGL3- IAP92L23neo^TNF^) together with pT7-MOV10 or pT7-Luc. Cells were selected with G418 for 12-14 days to measure retrotransposition frequency and then fixed and stained with Giemsa. **(B)** MOV10 overexpression has no affect on *neo* expression or selection. HeLa cells were co-transfected with a pcDNA3.1 control vector containing a neomycin resistance cassette (pcDNA3.1-*neo*) together with pT7-MOV10 or pT7-Luc*.* The cells were G418 selected and the colonies were quantified as described in panel (A). Results are normalised to the pLuc control, which is set at 100%. For (A) a single control bar set at 100% is graphed for simplicity. Values are the mean ± SD of 3 independent experiments**.**

Established cell culture-based retrotransposition assays were used to study these retroelements [44-46]. Briefly, HeLa cells were transfected with expression plasmids for human LINE-1 (pJM101/L1.3) [47], human Alu (pAlu-neo^Tet^) [48] or mouse IAP (pGL3-IAP92L23neo^TNF^) [49] all of which contain an antisense neomycin resistance gene cassette (*neo*) in the 3′UTR driven by its own promoter and disrupted by an intron. *Neo* expression occurs only after a full retrotransposition event: specifically, transcription of the retroelement RNA, removal of the intron by splicing, translation of the proteins, reverse transcription and then integration of the cDNA into the host cell genome, allowing for enumeration of retrotransposition by counting G418-resistant colonies. The Alu element is dependent on LINE-1 enzymes for replication; therefore, to measure Alu element retrotransposition, the cells were also co-transfected with a plasmid encoding the LINE-1 ORF2 protein (pCEP-ORF2) [48], which encodes the LINE-1 endonuclease and reverse transcriptase enzymatic activities. Either pMOV10 or the pLuc control was co-transfected to determine the effect of MOV10 overexpression on the replication of these endogenous retroelements. Similar to the observations made with exogenous retroviruses (Figure 1), overexpression of MOV10 decreased human LINE-1, Alu and mouse IAP retrotransposition by over 90% when compared with the pLuc control (Figure 2A).

As a control, HeLa cells were also transfected with a pcDNA3.1 vector that contains a neomycin resistance expression cassette (pcDNA3.1-*neo*) to ensure that MOV10 overexpression did not affect *neo* expression or selection directly. The cultures were G418-selected and the colonies were counted as described for the retrotransposition assays, with similar numbers of colonies seen in the context of MOV10 overexpression as for the pLuc control (Figure 2B). Therefore, MOV10 overexpression inhibits the propagation of multiple endogenous retroelements.

### Silencing endogenous MOV10 does not affect the production of infectious retroviral particles

**Figure 3:**
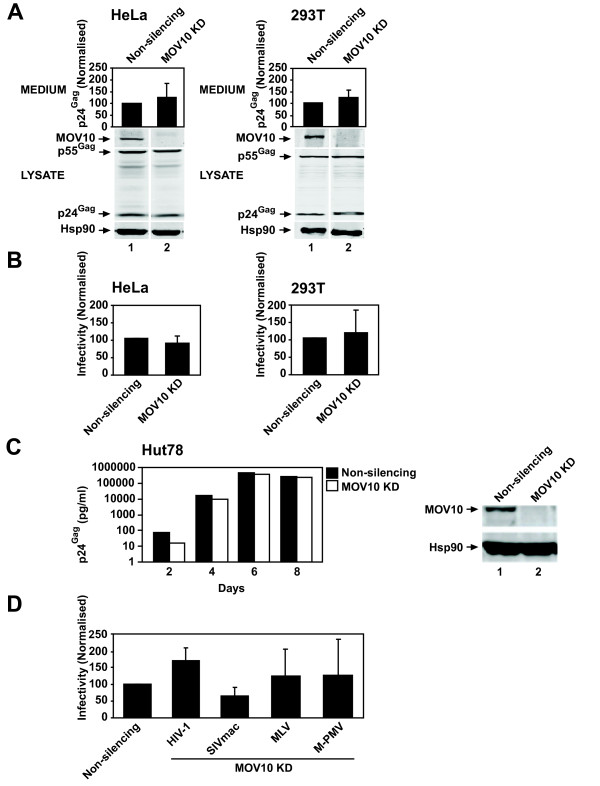
**Silencing endogenous MOV10 has no significant effect on the production of infectious retrovirus particles for a panel of exogenous retroviruses. (A)** Depletion of endogenous MOV10 has no effect on HIV-1 virus production. Stable MOV10 KD cells were produced by transducing HeLa or 293T cells with lentiviral vectors expressing either a non-silencing control shRNA or a MOV10-specific shRNA. HeLa or 293T non-silencing control and MOV10 KD cells were infected with VSV-G pseudotyped HIV-1_NL4-3_. Virus concentration in the medium was determined as described in Figure 1A. Cell lysates were analysed by immunoblotting with anti-p24^Gag^, anti-Hsp90 or anti-MOV10 antibodies, the latter of which was used to verify the MOV10 KD. (HeLa virus production p = 0.0611, 293T virus production p = 0.2007). **(B)** Silencing of endogenous MOV10 has no effect on HIV-1 virion infectivity. Virion infectivity was determined as described in Figure 1B. (HeLa infectivity p = 0.3080, 293T infectivity p = 0.4812). **(C)** Depleting endogenous MOV10 has no effect on spreading HIV-1 replication. Hut78 non-silencing control or MOV10 KD cells were infected with equal amounts of HIV-1_NL4-3_ and passaged every 2 days. Medium was harvested on days 2, 4, 6 and 8 and virus production was determined as described in Figure 1A. Cell lysates were analysed by immunoblotting with anti-MOV10 and anti-Hsp90 antibodies. **(D)** MOV10 silencing has no effect on the production of infectious SIVmac, MLV and M-PMV particles. 293T non-silencing control or MOV10 KD stable cells were transfected as described in Figure 1C for the production of HIV-1, SIVmac, MLV and M-PMV particles. Infectivity was determined as described in Figure 1C. (HIV-1 p = 0.1358, SIVmac p = 0.1040, MLV p = 0.4907, M- PMV p = 0.4919). For (A), (B) and (D) results are normalised to the non-silencing control, which is set at 100%. For (D) a single control bar set at 100% is graphed for simplicity. Values are the mean ± SD of 7 independent experiments for (A) and (B) or 3 independent experiments for (D). The data were analysed with an unpaired one- tailed *t* test**.**

We next determined the effect of depleting endogenous MOV10 on HIV-1 production and infectivity in the context of one full cycle of viral replication. Stable HeLa and 293T non-silencing control and MOV10 knockdown (KD) cell lines were produced by transducing HeLa or 293T cells with lentiviral vectors expressing either a non-silencing control shRNA or a MOV10-specific shRNA, which reduced MOV10 protein steady-state abundance to undetectable levels when compared with the non-silencing control cells (Figure 3A). The depletion of endogenous MOV10 did not affect the growth rate of these cells (data not shown). HeLa or 293T non-silencing control and MOV10 KD cell lines were infected with equal amounts of VSV-G pseudotyped HIV-1_NL4-3_ and virion production and infectivity were determined. Depletion of endogenous MOV10 showed no significant effect on the amount (Figure 3A) or infectivity (Figure 3B) of virions produced in comparison to the non-silencing control in both HeLa and 293T cells. Similar experiments were performed with comparable results using a second, unrelated MOV10-specific shRNA (data not shown). To determine whether depletion of endogenous MOV10 affects multiple rounds of HIV-1 replication, we infected stable non-silencing control or MOV10 KD Hut78 T cells with equal amounts of HIV-_1NL4-3_ and determined the effect on virus production. Consistent with the single-cycle infectivity assays, silencing of MOV10 had no effect on spreading HIV-1 replication (Figure 3C).

To determine whether endogenous MOV10 regulates the production of infectious SIVmac, MLV or M-PMV, virions were produced as described above in 293T non-silencing control or MOV10 KD cells and the effect on infectious particle production was determined. Depletion of endogenous MOV10 had no significant effect on the production of HIV-1, SIVmac, MLV or M-PMV infectious particles (Figure 3D). Although we cannot rule out the possibility that undetectable levels of residual MOV10 in our KD cultures are still functional, these data strongly suggest that endogenous levels of MOV10 do not control the replication of exogenous retroviruses.

### Depletion of endogenous MOV10 specifically enhances the retrotransposition of endogenous retroelements

**Figure 4:**
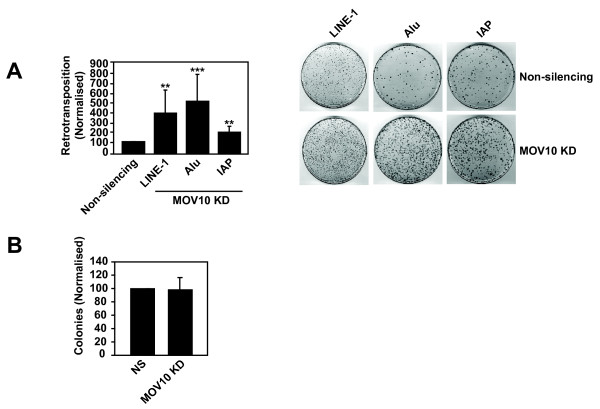
**Depletion of endogenous MOV10 significantly enhances the retrotransposition of endogenous retroelements. (A)** Silencing of endogenous MOV10 significantly enhances the retrotransposition of LINE-1, Alu and IAP. As described in Figure 2A, HeLa non-silencing control or MOV10 KD stable cell lines were transfected with retroelement expression plasmids and retrotransposition was quantified. (LINE-1 **p ≤ 0.0056, Alu ***p ≤ 0.0005, IAP **p ≤ 0.0096). **(B)** MOV10 silencing does not effect *neo* expression or selection directly. As described in Figure 2B, HeLa non-silencing control or MOV10 KD cells were transfected with the pcDNA3.1-*neo* control vector and colonies were quantified. Results are normalised to the non-silencing control, which is set at 100%. For (A) a single control bar set at 100% is graphed for simplicity. Values are the mean ± SD of 3 independent experiments. The data were analysed with an unpaired one-tailed *t* test**.**

We next determined the effect of silencing endogenous MOV10 on LINE-1, Alu and IAP replication in the HeLa non-silencing control or MOV10 KD cells. In the absence of detectable MOV10, statistically significant 4-fold, 5-fold and 2-fold enhancements in retrotransposition frequencies were detected for LINE-1, Alu and IAP, respectively (Figure 4A). We also transfected HeLa non-silencing control or MOV10 KD cell lines with the pcDNA3.1-*neo* control plasmid and obtained similar number of colonies with the non- silencing control and MOV10 KD cell lines verifying that silencing of endogenous MOV10 does not effect *neo* expression and selection directly, and also has no effect on transfection efficiency (Figure 4B).

To confirm that the increase in endogenous retroelement replication was due to the depletion of endogenous MOV10 and not an unanticipated off-target effect, we constructed a silencing resistant MOV10 vector, pMOV10-R, by introducing silent mutations that prevented recognition by the shRNA. The antiviral activity of MOV10 was unaffected by these mutations as overexpression of pMOV10-R inhibited the production of infectious MLV and M-PMV virions to a similar magnitude as parental pMOV10 (data not shown). The shRNA resistance of pMOV10-R was confirmed by titrating sensitive pMOV10 and resistant pMOV10-R into the HeLa MOV10 KD cells, and analysing cell lysates by immunoblotting ( Additional file 1A). Results showed that the levels of MOV10 encoded by pMOV10-R were elevated in the KD cells relative to those seen with the parental pMOV10 vector ( Additional file 1A, compare lanes 5 and 6 with 11 and 12). Next, we transfected MOV10 KD cells with pMOV10-R to test the functional consequence of restoring MOV10 expression, and found that the suppression of LINE-1 retrotransposition was re-established ( Additional file 1B).

Thus, endogenous human MOV10 specifically represses the propagation of intracellular retroelements.

### MOV10 is not necessary for miRNA or siRNA-mediated mRNA silencing

MOV10 interacts with the Argonaute proteins, which are central effector components of the RISC, and has been reported to be necessary for siRNA-mediated mRNA silencing by an endogenous miRNA [27]. To determine whether MOV10 is necessary for small RNA-mediated RNA silencing, which is one possible mechanism by which MOV10 may control the replication of endogenous retroelements, we initially tested the requirement of MOV10 for miRNA-mediated mRNA repression. HeLa non-silencing control or MOV10 KD cell lines were transfected with either a firefly (FF) luciferase reporter construct containing four copies of the endogenous let-7 miRNA binding site (FF4LCS; let-7 WT) or a negative control carrying mutations in the target seed region of the let-7 binding sites (FFr4mLCS; let-7 mutant), together with a control plasmid expressing renilla luciferase (pRenilla) [50]. Cells were lysed and the relative FF luciferase and renilla luciferase activities were determined. FF luciferase activity was normalised to the renilla luciferase activity to control for transfection efficiency.

**Figure 5:**
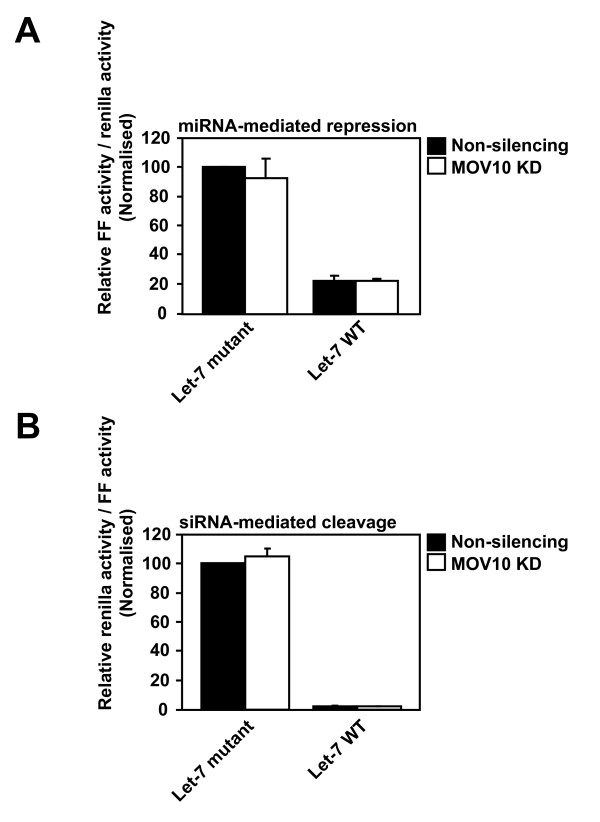
**MOV10 is not necessary for miRNA or siRNA-mediated mRNA silencing**. **(A)** MOV10 is not necessary for miRNA-mediated mRNA repression. HeLa non-silencing control or MOV10 KD cells were co-transfected with either FF4LCS (let-7 WT) or FFr4mLCS (let-7 mutant) together with pRenilla. Relative luciferase activities were measured using a Dual-Luciferase® Reporter Assay System and FF luciferase activity was normalised to the renilla luciferase activity. **(B)** MOV10 is not required for siRNA-mediated mRNA cleavage. HeLa non-silencing control or MOV10 KD cells were transfected with either psi-CHECK2-let-7X3 (let-7 WT) or psi-CHECK2-let-7X3m (let-7 mutant). Luciferase activities were measured as described in panel (A) and renilla luciferase activity was normalised to FF luciferase activity. Results are normalised to the non-silencing control, which is set at 100%. Values are the mean ± SD of 3 independent experiments**.**

As expected, the let-7 WT luciferase activity was repressed ~5-fold compared to the let-7 mutant luciferase activity in the non-silencing control cells (Figure 5A). A similar 5-fold repression in let-7 WT luciferase activity relative to the let-7 mutant luciferase activity was observed in the MOV10 KD cells suggesting that MOV10 is not required for endogenous let-7 miRNA-mediated mRNA repression in HeLa cells (Figure 5A). As a control for this assay, we also knocked down DICER-1, which is an RNase III enzyme essential for miRNA biogenesis, and co-transfected DICER-1 KD or non-silencing control cells with the FF luciferase reporter constructs and pRenilla. As expected, and confirming the validity of our approach, a 70% decrease in DICER-1 mRNA expression resulted in a partial derepression of the let-7 miRNA activity ( Additional file 2).

To determine whether MOV10 is required for siRNA-mediated mRNA silencing as previously reported [27], HeLa non-silencing control or MOV10 KD cells were transfected with renilla luciferase reporter constructs containing three copies of perfectly complementary let-7 miRNA binding sites (psi-CHECK2-let-7X3; let-7 WT) or mutated let-7 binding sites (psi-CHECK2-let-7X3m; let-7 mutant) [51]. The perfect complementarity between endogenous let-7 miRNA and the reporter mRNA promotes siRNA-mediated cleavage instead of miRNA-mediated repression. Both let-7 WT and let-7 mutant constructs also expressed FF luciferase from a second promoter and the renilla luciferase activity was normalised to FF luciferase activity to control for transfection efficiency. In the non-silencing control cells, let-7 WT luciferase activity was repressed by ~50-fold relative to the let-7 mutant luciferase activity, and this level of repression was maintained in the MOV10 KD cells (Figure 5B). These results imply that, at least in HeLa cells, MOV10 is not essential for miRNA or siRNA-mediated mRNA silencing.

### MOV10 is dispensable for the restriction of LINE-1 or HIV-1 replication by APOBEC3 proteins

Overexpression experiments have previously shown that a number of APOBEC3 proteins such as APOBEC3A (A3A), APOBEC3B (A3B) and A3G inhibit the retrotransposition of endogenous retroelements [6-8,10] and the depletion of endogenous A3B increases the replication of human LINE-1 in HeLa and human embryonic stem cells [10]. Since MOV10 was identified as an APOBEC3-interacting protein, we determined whether A3A, A3B or A3G require MOV10 for the restriction of LINE-1. HeLa non-silencing control or MOV10 KD cells were co-transfected with either pA3A, pA3B, pA3G or a pGFP control (pCMV4-HA tagged A3A, A3B, A3G or GFP) [52] together with pLINE-1 as described. A3A completely inhibited LINE-1 retrotransposition both in the presence or absence of MOV10 (Additional file 3A). A3B restricted LINE-1 replication in the non-silencing control and MOV10 KD cells by 70% and 66%, respectively, while A3G inhibited LINE-1 retrotransposition by 50% and 56%, respectively, relative to the non-silencing control and MOV10 KD pGFP controls (Additional file 3A). Therefore, A3A, A3B and A3G do not require MOV10 for the inhibition of LINE-1 mobilisation.

Similarly, we also tested whether MOV10 is required for A3G antiviral activity by co-transfecting HeLa non-silencing control and MOV10 KD cells with pA3G or a pGFP control together with a plasmid expressing a vif-deficient HIV-1 provirus (pHIV-1_IIIB_/Δvif) [52]. Virion infectivity was determined using the TZM-bl reporter cell line and results showed that A3G still inhibited HIV-1 infectivity in the absence of endogenous MOV10, suggesting that MOV10 is not required for A3G antiviral activity (Additional file 3B).

## Discussion

Host cell restriction factors inhibit the replication of a diverse range of exogenous retroviruses and endogenous retroelements. Identifying the full complement of these proteins is necessary to understand the capacity of the host to regulate and control these genetic parasites. MOV10 has been reported to modulate the replication of a variety of RNA viruses including HCV, HDV and VSV [18-20]. Here, we analysed whether MOV10 controls the replication of exogenous retroviruses and endogenous retroelements.

Our results show that MOV10 overexpression restricts the production of infectious retrovirus particles (Figure 1). This broadly agrees with previously published reports [30-32], and extends the finding to the betaretrovirus M-PMV (Figure 1C and D). Similar to Furtak et al., [31] we observe a greater decrease in HIV-1 virion infectivity compared to virion production, and this is more obvious in 293T cells (Figure 1A and B). We also observe a modest decrease in cellular HIV-1 Gag abundance and processing similar to that reported by Burdick et al. [30], as well as a more noticeable decrease in cellular Gag abundance and processing for M-PMV (Figure 1A and D). As virion assembly is a cooperative process, decreases in total intracellular Gag abundance may account for the reductions in Gag processing [53]. MOV10 can also be packaged into budding HIV-1 virions [30,32-34] and, interestingly, the overexpression of MOV10 in HIV-1 producing cells decreases the accumulation of early reverse transcription products in target cells (data not shown) [31,32]. The mechanism(s) underlying the defects in virion production and reverse transcription are unclear, though the generality of these observations across retroviral genera suggests a common mode of action.

As described above, three groups have analysed the role of endogenous MOV10 in HIV-1 replication, but have reported variable results [30-32]. It was important for us to test the effect of depleting endogenous MOV10 on HIV-1 replication, and we extended this to include a panel of exogenous retroviruses. Contrary to the previous reports, we observe that depletion of endogenous MOV10 has no affect on the production of infectious retroviral particles or spreading HIV-1 replication (Figure 3). This result is similar to that reported recently for foamy virus, a distantly related retrovirus belonging to the spumaretrovirus subfamily, where knockdown of MOV10 had no effect on viral replication [54]. In sum, while it appears that endogenous levels of MOV10 do not restrict retroviral replication, we speculate that the results of overexpression studies implicate MOV10 as a component of a pathway or multiple pathways that exogenous retroviruses encounter. MOV10 has also been reported to be a type I interferon-stimulated gene [19], but whether interferon or other cytokines can stimulate sufficient levels of MOV10 protein to impact exogenous retrovirus infections is not yet known.

The MOV10 ortholog Armitage is required for the repression of endogenous mobile elements in both germ cells and somatic cells in *Drosophila melanogaster*[22,25,26]. Similarly, the MOV10 paralog, MOV10L1, has been shown to be necessary for the silencing of endogenous retrotransposons in the germ line of male mice [23,24]. Therefore, we analysed whether human MOV10 could inhibit endogenous retroelements. Similar to its effect on exogenous retroviruses, the overexpression of MOV10 potently inhibits the transposition of the human endogenous retrotransposons LINE-1 and Alu as well as the mouse endogenous retrovirus IAP (Figure 2A). Unlike the exogenous retroviruses, however, the depletion of endogenous MOV10 significantly enhances the replication of LINE-1, Alu and IAP (Figure 4A), which in the case of LINE-1 can be reversed by restoration of MOV10 expression with an shRNA-resistant version of MOV10 (Additional file 1). The mechanism by which MOV10 controls these LTR and non-LTR endogenous retroelements is unknown, but previous studies have shown that *Dicer1* knockout mouse embryonic stem cells have increased levels of LINE-1 and IAP transcripts [55]. Furthermore, Yang et al., [56] showed that human LINE-1 bidirectional transcripts produced from the LINE-1 sense and antisense promoters (ASP) are processed to yield LINE-1 specific endogenous siRNAs that suppress LINE-1 retrotransposition by an RNAi mechanism.

Although it has been reported that MOV10 associates with the RISC pathway and is necessary for siRNA-mediated silencing of target mRNAs [27], our findings to date using reporter constructs indicate that MOV10 is not absolutely required for miRNA or siRNA-mediated mRNA silencing in cultured cells (Figure 5A and B); therefore, whether this is a mechanism by which MOV10 could regulate endogenous retroelements is unclear. MOV10 also localises to mRNA processing bodies (PBs) [27,29], which are cytoplasmic sites involved in the storage and decay of translationally repressed RNA species, and it has recently been reported that silencing of the PB-associated proteins DDX6 and 4E-T increases IAP transcript levels and promotes IAP retrotransposition [57]. Taking this into consideration, we are currently investigating the pathway/mechanism by which MOV10 regulates retroelement mobility.

Human MOV10 is expressed in a wide range of adult tissues including the heart, lungs, liver, testes and ovaries with the highest transcript levels detected in the adult brain including the hippocampus and caudate nucleus [58]. Intriguingly, recent studies have shown that LINE-1 transcripts are expressed in most human somatic tissues as opposed to being confined to the germ line [3]. Furthermore, active LINE-1, Alu and SVA element retrotranspositions in the human hippocampus and caudate nucleus have been reported to contribute to the genetic mosaicism of the human brain that may underlie both normal and abnormal neurobiological processes [2,4]. Based on our observations that endogenous MOV10 regulates LINE-1 and Alu replication (Figure 4A), it will be interesting to determine whether human MOV10 may be involved in the modulation of somatic retrotransposition and contribute to the control of retrotransposition-mediated genetic variation.

## Conclusion

MOV10 overexpression potently restricts the replication of a broad range of exogenous and endogenous retroelements. Silencing endogenous MOV10 has no effect on the replication of exogenous retroviruses, but it significantly enhances the retrotransposition of endogenous retroelements. We hypothesise that MOV10 may contribute to the regulation of endogenous retroelement mobilisation in somatic cells.

## Methods

### Cell culture, MOV10 RNAi and plasmids

Human HeLa and 293T cells were cultured in Dulbecco's modified Eagle's medium while Hut78 cells were cultured in RPMI. Both types of media were supplemented with 10% fetal bovine serum plus penicillin-streptomycin and L-glutamine. 293T cells were co-transfected with lentiviral vectors expressing either a non-silencing control or MOV10-specific shRNAmir in the miR-30 context containing a puromycin resistance gene (GIPZ Lentiviral shRNAmir, Open Biosystems V2LHS_201304), together with the HIV-1 p8.91 packaging plasmid and pVSV-G (see plasmids below). HeLa, 293T and Hut78 cells were transduced with the recombinant lentiviral stocks and stably transduced cells were selected with puromycin treatment.

The pT7-MOV10 and pT7-Luc plasmids were constructed by cloning XbaI-BamHI digested full-length MOV10 and FF luciferase PCR products into the pCGTHCF_FL_T7 expression vector that contains two 5′-T7-epitope tags [59]. The pMOV10-R plasmid was constructed by introducing six silent mutations into the MOV10-specific shRNA target sequence (nucleotides 342 to 363) by overlapping PCR (Primers: Forward 5′ TTTATGACAGGGC***C***GA***A***TA***C***CT***C***CA***C***GG***A***AAACATGGTGTGG 3′, Reverse 5′ CCACACCATGTTT***T***CC***G***TG***G***AG***G***TA***T***TC***G***GCCCTGTCATAAA 3′) and cloning the XbaI-XmaI digested PCR product into a similarly digested pT7-MOV10 vector. The HIV-1_NL4-3_ strain provirus was used for this study [35]. Plasmids for exogenous retrovirus and endogenous retroelement experiments have been described previously: pVSV-G [37]; HIV-1, p8.91 and pCSGW [38,39]; SIVmac, pSIV3+ and pSIV-RMES4 [36]; MLV, pNCS/FLAG and pMSCV/Tat [40,41] M-PMV, pMTΔE [42]; LINE-1, pJM101/L1.3 [47]; Alu, pAlu-neo^Tet^ and pCEP-ORF2 [48]; IAP, pGL3- IAP92L23neo^TNF^[49]. Plasmids for the luciferase assays were described previously: FF4LCS, FFr4mLCS, pRenilla, psi-CHECK2-let-7X3 and psi-CHECK2-let-7X3m [50,51].

### Virus production and infectivity assays

For wild-type HIV-1 virus production, parental HeLa or 293T cells (2 x 10^5^ cells) were transfected with 0.5 μg of a plasmid expressing the full-length HIV-1_NL4-3_ strain provirus (pHIV-1_NL4-3_) using either FuGENE 6 (Roche) according to manufacturer's instructions at a 3 μl FuGENE to 1 μg DNA ratio for the HeLa cells, or 16 μl (1 mg/ml) PEI (per well of a 6-well dish) for the 293T cells. For MOV10 overexpression experiments, pHIV-1_NL4-3_ was co-transfected with the indicated concentration of pT7-MOV10 and the appropriate concentration of the pT7-Luc control plasmid to ensure equivalent amounts of DNA in all transfections. For HIV-1 and SIVmac lentiviral vector production, parental 293T cells or 293T non-silencing control and MOV10 KD cells were co-transfected as described with 1 μg p8.91, 1 μg pCSGW and 0.5 μg pVSV-G, or 1 μg pSIV3+, 1 μg pSIV-RMES4 and 0.5 μg pVSV-G, respectively. MLV and M-PMV virions were produced by co-transfecting parental 293T cells or non-silencing control and MOV10 KD cells as described with 0.2 μg pNCS/FLAG, 0.2 μg pMSCV/Tat and 0.1 μg pVSV-G, or 1 μg pMTΔE and 0.5 μg pVSV-G, respectively. Plasmids for lentiviral vector or MLV and M-PMV virion production were co-transfected with 0.5 μg pT7-MOV10 or pT7-Luc for MOV10 overexpression experiments. Cells were lysed ~40 h post-transfection and virus particles were filtered through a 0.45 μM filter. The concentration of HIV-1 p24^Gag^ in the supernatant was quantified by a p24^Gag^ enzyme-linked immunosorbent assay (ELISA) (Perkin-Elmer).

For HIV-1 infectivity, the TZM-bl reporter cell line (1 x 10^5^ cells) expressing a HIV-1 Tat inducible β-gal reporter gene was challenged with equal amounts of virus normalised by the p24^Gag^ concentration. Cells were lysed ~24 h post-infection and β-gal activity was determined using the Galacto-Star^TM^ System (Applied Biosystems) according to the manufacturer's instructions. For HIV-1 and SIVmac lentiviral vector infectivity, 293T cells (1 x 10^5^ cells) were challenged with equal amounts of vector- containing medium and infectivity was determined ~24 h post-infection by measuring the percentage of GFP-positive 293T cells using a FACS Canto II Flow Cytometry System (BD Biosciences). MLV and M-PMV virion infectivity was determined by infecting TZM-bl cells (1 x 10^5^ cells) with equal amounts of virus-containing medium and infectivity was determined as described for the wild-type HIV-1 experiments.

### HIV-1 infection of producer cells and spreading infection

For endogenous MOV10 silencing experiments, HeLa or 293T non-silencing control and MOV10 KD cells (2 x 10^5^ cells) were infected with equal amounts of VSV-G pseudotyped wild type HIV-1_NL4-3_ virus normalised by the p24^Gag^ concentration (25 ngs) in a total of 1 ml medium (6-well dish). The cells were washed 4 h later and 2 mls of fresh medium was replaced. For spreading replication, non-silencing control or MOV10 KD Hut78 cells (1 x 10^6^ cells) were infected with equal amounts of virus normalised by the p24^Gag^ concentration (100 ngs) and cells were passaged every 2 days. Medium was harvested on days 2, 4, 6 and 8 and virus production was measured by p24^Gag^ ELISA.

### Immunoblotting

Cells were lysed in radioimmunoprecipitation assay (RIPA) buffer (10 mM Tris-HCl pH 7.5, 150 mM NaCl, 1 mM EDTA, 0.1% SDS, 1% Triton X-100, 1% sodium deoxycholate) and filtered virions were pelleted through a 20% sucrose cushion and lysed. Proteins were resolved by SDS-PAGE and transferred to a nitrocellulose membrane for immunoblotting. HIV-1 precursor p55^Gag^ and processed p24^Gag^ were detected using a mouse anti-p24^Gag^ antibody [60]. M-PMV precursor p78^Gag^ and processed p27^Gag^ were detected using goat anti-p27^Gag^ antisera (78 S-136, Microbiological Association). T7-tagged MOV10 and Hsp90 were detected with mouse anti-T7 (Novagen) and rabbit anti-Hsp90 (Santa Cruz Biotechnology) antibodies, respectively. Endogenous MOV10 was detected with a rabbit anti-MOV10 antibody (Proteintech). Secondary IRdye800 conjugated antibodies (Li-Cor Biosciences) were used for quantitative immunoblotting with the Odyssey infrared scanner (Li-Cor Biosciences).

### Retrotransposition assays

For LINE-1, Alu and IAP retrotransposition assays parental HeLa cells or HeLa non-silencing control and MOV10 KD cells (2 x 10^5^ cells) were co-transfected as described with either 1.5 μg pJM101/L1.3, 1 μg pAlu-neo^Tet^ plus 0.5 μg pCEP-ORF2 or 1.5 μg pGL3-IAP92L23neo^TNF^, respectively. Plasmids were co-transfected with 1 μg pT7-MOV10 or pT7-Luc for MOV10 overexpression experiments. Cells were G418 selected (1 mg/ml) 2 days post-transfection. At ~12-15 days post-transfection, the cells were fixed in 4% paraformaldehyde and colonies were stained with 0.4% Giemsa (Sigma) for counting. For control pcDNA3.1-*neo* experiments cells were transfected with 0.3 μg of a pcDNA3.1 empty vector containing a neomycin resistance cassette, and the assay was performed similarly to the retrotransposition assays.

### Luciferase assays

For the miRNA assays, HeLa non-silencing control or MOV10 KD cells (1 x 10^5^ cells) were co-transfected as described with either 0.1 μg FF4LCS or FFr4mLCS together with 0.1 μg pRenilla. For the siRNA assays, the cells were transfected with 0.1 μg psi-CHECK2-let-7X3 or psi-CHECK2-let-7X3m. Cells were lysed ~24 h post- transfection. Relative luciferase activities were measured using a Dual-Luciferase® Reporter Assay System (Promega) according to the manufacturer's instructions.

## Competing interests

The authors declare that they have no competing interests.

## Authors’ contributions

SA conducted the experiments, performed the analyses and interpretation of the data, and wrote the manuscript. CMS and MHM helped write the manuscript. CMS, NMS and MHM conceived the study and contributed to data interpretation, and MHM supervised the project. SMW provided experimental tools for the study. NMS and SMW also contributed to the drafting of the manuscript. All authors read and approved the final manuscript.

## Supplementary Material

Additional file 1 **Restoration of MOV10 expression rescues the control of LINE-1 retrotransposition.(A)** HeLa MOV10 KD cells were transfected with increasing concentrations of >pT7-MOV10 or pT7-MOV10-R. Cells were analysed by immunoblotting with anti-MOV10, anti-T7 and anti-Hsp90 antibodies. **(B)** HeLa non-silencing control or MOV10 KD cells were co-transfected with pLINE-1 (pJM101/L1.3) together with pT7-MOV10-R or pT7-Luc at the indicated concentrations, following which the cultures were G418 selected and colonies were counted to measure the retrotransposition frequency. Cell lysates were analysed by immunoblotting with anti-MOV10, anti-T7 and anti-Hsp90 antibodies. For (B) results are normalised to the non-silencing control, which is set at 100%. Values are the mean ± SD of 3 independent experiments. Click here for file

Additional file 2 **Knockdown of DICER-1 relieves miRNA-mediated mRNA repression.** HeLa cells were transfected with non-silencing control or DICER-1-specific siRNAs to produce non-silencing control or DICER-1 KD cells, respectively. These cells were co-transfected with either FF4LCS (let-7 WT) or FFr4mLCS (let-7 mutant) together with pRenilla. The relative luciferase activities were measured using a Dual-Luciferase® Reporter Assay System. FF luciferase activity was normalised to renilla luciferase activity. Click here for file

Additional file 3 **MOV10 is not required for restriction of LINE-1 or HIV-1 infection by APOBEC3 proteins.(A)** HeLa non-silencing control or MOV10 KD cells were co-transfected with pLINE-1 (pJM101/L1.3) and pCMV4-HA tagged A3A, A3B, A3G or a GFP control. Cells were G418 selected and colonies were quantified to determine the retrotransposition frequency. **(B)** HeLa non-silencing control or MOV10 KD cells were co-transfected with pHIV-1_IIIB_/Δvif and either pA3G or pGFP. Infectivity was determined by infecting TZM-bl cells with equal amounts of virus normalised by the p24^Gag^ concentration. For (A) results are normalised to the non-silencing control, which is set at 100%. Values are the mean ± SD of 3 independent experiments. Click here for file
