# Supplementary figures and images for: Endogenous MOV10 inhibits the retrotransposition of endogenous retroelements but not the replication of exogenous retroviruses
Source: Retrovirology. 2012 Jun 22;9:53. doi: 10.1186/1742-4690-9-53 (PMC3408377; doi:10.1186/1742-4690-9-53)

**A**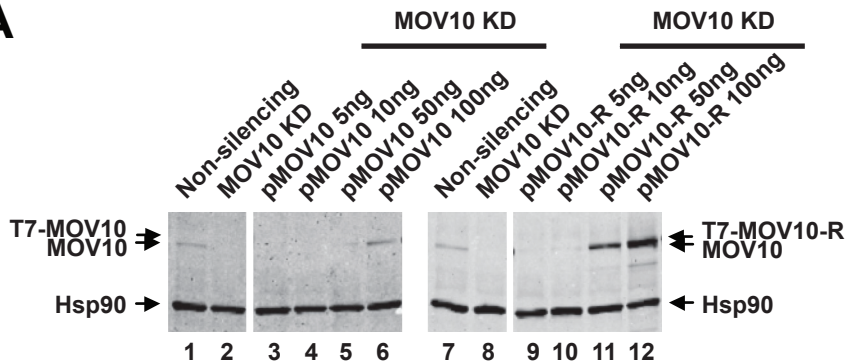**B**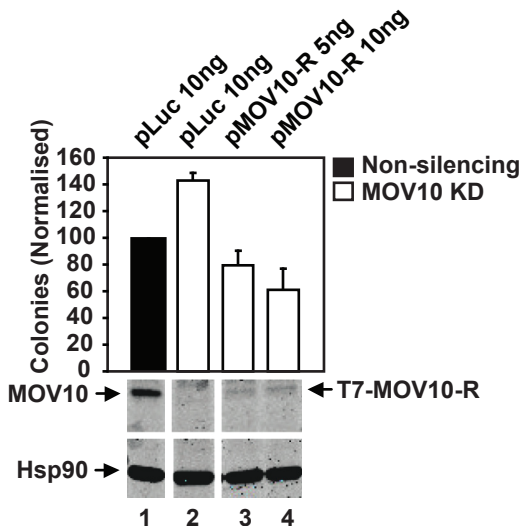

Supplement: Additional file 1 — Restoration of MOV10 expression rescues the control of LINE-1 retrotransposition.(A) HeLa MOV10 KD cells were transfected with increasing concentrations of >pT7-MOV10 or pT7-MOV10-R. Cells were analysed by immunoblotting with anti-MOV10, anti-T7 and anti-Hsp90 antibodies. (B) HeLa non-silencing control or MOV10 KD cells were co-transfected with pLINE-1 (pJM101/L1.3) together with pT7-MOV10-R or pT7-Luc at the indicated concentrations, following which the cultures were G418 selected and colonies were counted to measure the retrotransposition frequency. Cell lysates were analysed by immunoblotting with anti-MOV10, anti-T7 and anti-Hsp90 antibodies. For (B) results are normalised to the non-silencing control, which is set at 100%. Values are the mean ± SD of 3 independent experiments. [file 1742-4690-9-53-S1.pdf]

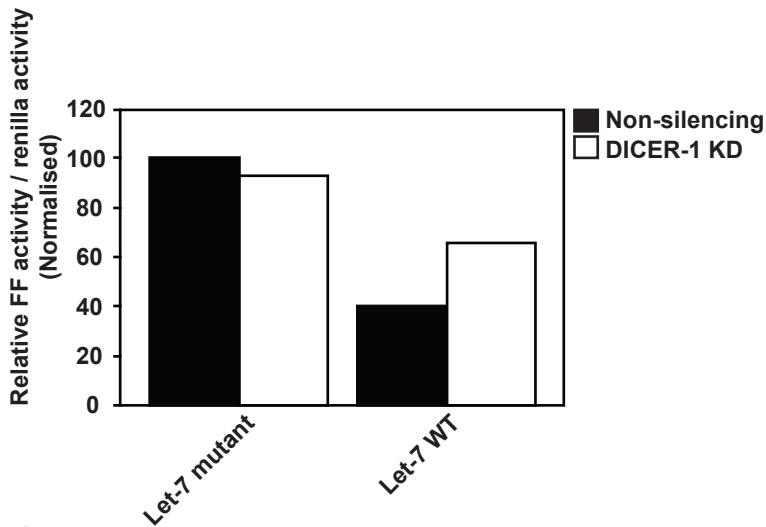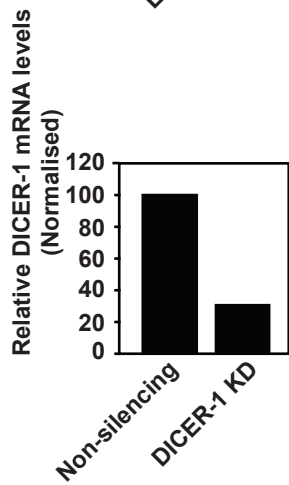

Supplement: Additional file 2 — Knockdown of DICER-1 relieves miRNA-mediated mRNA repression. HeLa cells were transfected with non-silencing control or DICER-1-specific siRNAs to produce non-silencing control or DICER-1 KD cells, respectively. These cells were co-transfected with either FF4LCS (let-7 WT) or FFr4mLCS (let-7 mutant) together with pRenilla. The relative luciferase activities were measured using a Dual-Luciferase® Reporter Assay System. FF luciferase activity was normalised to renilla luciferase activity. [file 1742-4690-9-53-S2.pdf]

**A**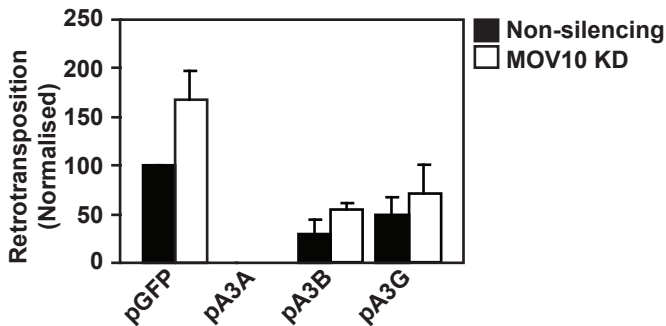**B**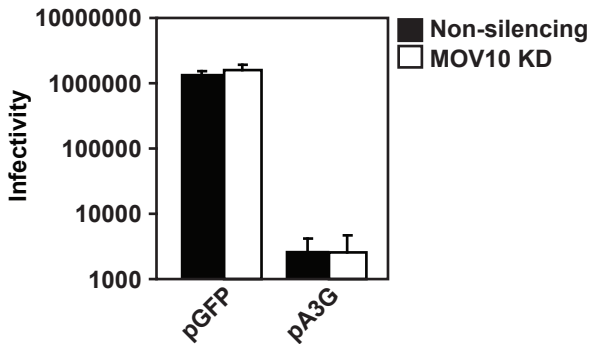

Supplement: Additional file 3 — MOV10 is not required for restriction of LINE-1 or HIV-1 infection by APOBEC3 proteins.(A) HeLa non-silencing control or MOV10 KD cells were co-transfected with pLINE-1 (pJM101/L1.3) and pCMV4-HA tagged A3A, A3B, A3G or a GFP control. Cells were G418 selected and colonies were quantified to determine the retrotransposition frequency. (B) HeLa non-silencing control or MOV10 KD cells were co-transfected with pHIV-1IIIB/Δvif and either pA3G or pGFP. Infectivity was determined by infecting TZM-bl cells with equal amounts of virus normalised by the p24Gag concentration. For (A) results are normalised to the non-silencing control, which is set at 100%. Values are the mean ± SD of 3 independent experiments. [file 1742-4690-9-53-S3.pdf]
